# Navigating cultural barriers: a qualitative study exploring clinicians’ experiences of providing mental health support for ethnic minority groups in England

**DOI:** 10.1186/s12913-026-14146-5

**Published:** 2026-02-07

**Authors:** Shireen Patel, Nikita R. Rattu, Priya Patel, Vibhore Prasad, Laurence Astill Wright, Camilla Babbage, Kapil Sayal, Madeleine J. Groom, Charlotte L. Hall

**Affiliations:** 1 NIHR Applied Research Collaboration, East Midlands, Institute of Mental Health, School of Medicine, Mental Health and Clinical Neurosciences, University of Nottingham, Innovation Park, Triumph Road, Nottingham, NG7 2TU UK; 2 Nottinghamshire Healthcare NHS Foundation Trust, Institute of Mental Health, Nottingham, UK; 3 NIHR MindTech MedTech Health Research Centre, Institute of Mental Health, School of Medicine, Mental Health and Clinical Neurosciences, University of Nottingham, Nottingham, UK; 4 Lifespan and Population Health, School of Medicine, University of Nottingham, Nottingham, UK; 5 King’s College London, London, UK; 6 Centre for Academic Mental Health, Population Health Sciences, University of Bristol, Bristol, UK; 7 Institute of Mental Health, School of Medicine, Mental Health and Clinical Neurosciences, University of Nottingham, Nottingham, UK; 8 NIHR Nottingham Biomedical Research Centre, Institute of Mental Health, University of Nottingham, Nottingham, UK

**Keywords:** Ethnic minority groups, Mental healthcare, Access, Barriers, Culture

## Abstract

**Background:**

Ethnic minority populations are increasing in England, however individuals from ethnic minority backgrounds are less likely to access mental health support compared with their White British counterparts, despite experiencing parallel but also unique risks of developing mental health difficulties. Understanding the barriers for ethnic monitory groups to accessing mental health services is therefore crucial. As clinicians are at the forefront of access to healthcare for all people, their perspectives on how ethnic minority groups are supported with their mental health are underexplored but may illuminate barriers to accessing mental health support. This study aimed to investigate clinicians’ experiences in supporting patients from ethnic minority backgrounds with mental health difficulties.

**Method:**

Fourteen clinicians participated in semi-structured interviews about their experiences of delivering mental healthcare to people from ethnic minority groups. The sample consisted of clinicians specialising in mental health (*n* = 9) and General Practitioners (*n* = 5) with 14.1 mean years of experience. Interviews explored clinicians’ approach to support, confidence when providing care, patient outlook on mental health and treatment, as well as service constraints. Data were analysed using reflexive thematic analysis.

**Results:**

Three main themes were identified: (1) Clinician personal factors impacting their approaches to supporting patients from ethnic minority groups; (2) Clinicians’ perspectives on how patients perceive mental health and mental health services; (3) Structural factors and level of training provided affects consultation and treatment. Perceived patient barriers to accessing support included cultural incompatibility, stigma and mistrust. Clinicians discussed the limited time and financial resources of services and how this restricted their capacity to make accommodations.

**Conclusions:**

This study highlights the importance of a comprehensive, multi-level strategy to overcome barriers to accessing mental health support among ethnic minority groups. Key recommendations include reviewing current training around culturally sensitive care, reforming systemic practices to foster inclusivity, and encouraging community engagement to reduce stigma and build trust in mental health services. Further research should focus on how cultural adaptations to services may improve engagement and explore the impact of intersectionality within and across individual ethnic groups. Such initiatives are crucial for achieving equitable mental health outcomes across diverse populations.

**Supplementary Information:**

The online version contains supplementary material available at 10.1186/s12913-026-14146-5.

## Background

Mental health difficulties represent a major global public health concern, with the World Health Organisation reporting substantial increases in the prevalence and severity of common mental disorders across all world regions. International research consistently shows that ethnic minority and migrant groups experience disproportionate exposure to psychosocial adversity, including discrimination, socioeconomic disadvantage, traumatic migration histories and marginalisation, all of which contribute to elevated vulnerability to mental health problems and poorer access to care [1]. Across world sub-regions such as South Asia, Africa and the Middle East, mental health clinicians frequently report challenges in supporting culturally diverse populations, including substantial communication barriers, low mental health literacy and differing cultural explanatory models for psychological distress [2, 3]. 

These challenges are also reflected across Europe, where studies indicate that clinicians often feel underprepared to manage the cultural complexities that arise when working with ethnic minority patients. Research from European countries suggests that clinicians encounter difficulties related to language, stigma, culturally shaped illness beliefs, limited culturally informed training, and mistrust of Western healthcare systems [4, 5]. However, because mental healthcare systems across Europe differ markedly in funding models, service structures and demographic composition [6], findings from these settings cannot be directly extrapolated to the patient and service context of England.

The UK population is culturally diverse with around 18% belonging to a non-white ethnic group. According to the UK Census, ethnic minority groups are defined as individuals who do not identify as White British. This includes people from a wide range of ethnic backgrounds, such as other White (e.g., Irish, Gypsy or Irish Traveller, and Other White groups), Asian or Asian British (e.g., Indian, Pakistani, Bangladeshi, Chinese) and Black, Black British, Caribbean or African [7]. The most prevalent ethnic minority groups in England, for example, are Indian (3.1%), Pakistani (2.7%) and Black African (2.5%) and these proportions are continuing to rise. For example, the percentage of people from Asian ethnic backgrounds in England and Wales has increased from 4.2 million (7.5%) in 2011 to 5.5 million (9.3%) in 2021 [7]. The prevalence of common mental disorders varies markedly in different ethnic minority groups. There is a higher prevalence of anxiety and depression in South Asian women (63.5% compared with 28.5% of white women) [8] and psychotic disorders in Afro-Caribbean men (3.1% compared with 0.2% of white men) [8]. Therefore, it is important that there is equal access to healthcare services for all, including mental health services [9]. However, ethnic minority groups are less likely to access mental health support compared with White British counterparts [10–12].

In England, mental healthcare is delivered across primary and secondary care. Primary care is often the first point of contact, where general practitioners (GPs) play a key role in identifying mental health concerns, providing initial support, and making referrals to specialist services. Secondary care involves specialist services for more complex needs, typically provided by clinical psychologists, psychiatrists, mental health nurses, and psychotherapists within community mental health teams or hospital settings. These professionals offer assessment, therapy, medication management, and ongoing care for individuals requiring specialist intervention.

Prompt access to mental health support is related to improved long-term outcomes and less severe, enduring difficulties ( [13, 14]) and is more cost-effective for health and social care systems ( [15, 16]). However, ethnic minority groups are less likely to access mental health support such as contacting their GP or being referred to specialist mental health services and may have greater difficulty accessing mental health services in the UK, compared to White British patients [17]. For example, Harwood et al. (2023) found that ethnic minority groups were less likely than White British people to self-refer themselves to the UK National Health Service (NHS) funded Improving Access to Psychological Therapies (IAPT), now known as NHS Talking Therapies, a programme designed to improve delivery of and access to evidence-based mental health services. Other research has indicated that IAPT practitioners may not be sufficiently trained in cultural competency ( [18, 19]). These findings highlight disparities in access to mental healthcare in England based on ethnicity.

Factors related to inequal access to mental healthcare based on ethnicity have been identified internationally, including greater financial difficulties, discrimination, language barriers, cultural dissonance, social isolation and unfamiliarity with the healthcare system [1]. However, existing research indicates that difficulties in accessing care may arise from multiple, interconnecting factors. Broadly, these could be categorised into patient-level factors and service-level factors. Notable patient-level factors have included observing that ethnic minority groups may have differing beliefs surrounding mental health, compared to Western norms, such as attributing their difficulties to spiritual and social causes more often [2, 3]. The Western conceptualisation of mental health defines mental health difficulties to have a biomedical or biopsychosocial basis, with treatment involving pharmacological and psychotherapeutic interventions [20]. Other patient-level factors include that ethnic minority groups may also be less aware of symptoms of mental health difficulties and thus are less able to identify them ( [21, 22]). They may also experience more stigma associated with mental health [23] including from their families and communities, preventing them from seeking support through mental health services. Ethnic minority groups may also have less trust in services [24] or have a negative outlook on treatment expectation, all of which are important barriers to accessing support ( [25, 26]). Experiences of racism and perceived unfair treatment can contribute to this mistrust in mental health services among ethnic minority groups in England [25], potentially affecting their willingness to access these services [24].

Research also distinguishes the role of service level factors in facilitating access for ethnic minority groups. Memon et al. (2016) found that language barriers, poor clinician-patient relationships, discrimination and cultural insensitivity also impeded access. Barriers at the service level can also include clinicians lacking cultural competence, i.e., the ability of clinicians and services to effectively deliver healthcare services that meet the social, cultural and linguistic needs of patients. Cultural competence in healthcare can include making adaptations [27]. An example of this is ethnic matching, which involves allocating a patient to a clinician with the same ethnic background [28], which has been found to increase patients’ positive regard of their mental health clinician [29].This is because clinicians’ understanding and interpretation of different cultures can significantly influence consultations, including how a patient’s history is interpreted and how management plans are developed [30]. Previous research has found that cultural competence training for clinicians in England is underdeveloped [31], not evidence-based and lacks theoretical grounding [28]. This may affect clinicians’ abilities to provide effective, culturally sensitive mental healthcare to patients from ethnic minority groups.

Conversely, appropriate general practitioner action (primary care), such as prompt referral and correct diagnosis, was found to be a facilitator to accessing treatment [26]. Furthermore, a systematic review found that adjusting clinician communication to consider cultural differences was important in enhancing commitment to mental health treatment for patients from ethnic minority backgrounds [5]. Examples of this included incorporating patients’ own views of mental health during discussions and treatment, simplifying language and openly discussing concerns patients may have in relation to their health and treatment.

Notably, most research in this area from a clinician perspective has been conducted in Europe [4] or the USA [31], but there has been comparatively less research in England [5, 18, 19]. Whilst some findings are likely to translate across country borders, given the systemic differences in healthcare funding and delivery [6] and differences in ethnic and cultural diversity, it is important to understand this within England. While recommendations for service improvements have previously been identified, ethnic disparities in access to mental health treatment continue to be prominent [17].

Earlier research has repeatedly called for more studies that examine clinician experiences, culturally informed care processes, culturally competent training and service-level barriers [5]. However, limited progress has been made in addressing these recommendations, resulting in persistent knowledge gaps surrounding how clinicians conceptualise and navigate cultural differences, how structural constraints shape their practice and how clinician ethnicity influences therapeutic relationships.To understand how best to support ethnic minority groups in England to receive timely and equal access to evidence-based mental health support, we aim to better understand clinicians’ experiences and perceptions of delivering mental healthcare to people from ethnic minority groups. By examining patient-level, clinician-level and system-level influences, this study provides an updated and contextually specific understanding of the barriers that clinicians perceive when supporting ethnic minority patients in England. This multi-level approach distinguishes the study from earlier work, which tended to focus on individual factors in isolation. Furthermore, the study responds directly to prior recommendations for greater research on cultural competence, adaptations to service delivery, and workforce diversity within England’s mental health system ( [32, 33]).

The aim of this research was to understand clinicians’ experiences of supporting adults from ethnic minority groups in England with mental health difficulties, in order to identify perceived barriers to accessing mental healthcare. By focusing on clinicians’ experiences of supporting ethnic minority groups within England’s NHS, the study contributes important and timely evidence to an area where significant knowledge gaps remain. Specifically, the research aims to answer two questions:

- What can clinicians tell us about how ethnic minorities are supported with their mental health by the National Health Service (NHS) in England?
- What barriers do clinicians perceive for ethnic minorities to accessing adequate mental health support?

## Method

### Study design

The current study was designed to explore the experiences of clinicians across multiple disciplines by using a reflexive qualitative approach to including general practitioners, clinical psychologists, psychiatrists and mental health practitioners. A qualitative approach on the epistemological basis that lived experience is composed of numerous related factors, guided by the Consolidated Criteria for Reporting Qualitative Research (COREQ) [34] was used (Supplementary Material 1). This research is situated within an interpretivist qualitative paradigm, as such, the study is guided by a theoretical orientation rather than a fixed theory, allowing findings to emerge inductively from participants’ narratives. Data was collected through semi-structured interviews with clinicians. This allowed for the holistic interpretation of interlinked factors from various levels, including patient, clinician and service factors, as opposed to investigating these as separate ‘variables’ [35]. These factors collectively came together to construct an overall understanding of how clinicians navigated providing mental health support to ethnic minority groups, and aided recognition of barriers to accessing effective mental healthcare.

### Participant recruitment

We sought to recruit clinicians with experience of supporting adults with mental health difficulties at any point during their contact with health services. Inclusion criteria were NHS clinicians in primary care such as GPs, or secondary care services, including clinical psychologists, psychiatrists, mental health practitioners, nurse specialists and therapists who had experience of assessing or treating adults from ethnic minority backgrounds experiencing mental health difficulties. Clinicians had to have been employed in their role for at least one year.

The selection of interview participants was based on convenience sampling to gain the views of a diverse range of perspectives across roles, service contexts and demographics. Participants self-selected following a recruitment call using a study advert, that stated that interviews were being conducted as part of a master’s research project. The advert was disseminated via digital newsletters produced by the Institute of Mental Health, a research centre at the University of Nottingham, in partnership with Nottinghamshire Healthcare NHS Foundation Trust. This newsletter was sent to approximately 2,300 affiliated staff once a month for three months. However, after only 3 clinicians expressed an interest to participate, the study team also approached established connections within research and clinical fields via email.

A target sample size of 10 to 15 participants was set, to appropriately deduce qualitative findings from interviews in health research [36].

Participants who expressed an interest in being interviewed were contacted via email by NRR, who provided information about the aims of the research and sent the Participant Information Sheet and consent form via email. Participants who provided written electronic consent to participate in the interview were interviewed. Recruitment of participants ended when information power or richness of data in relation to the aims of the study [37], had been reached.

### Procedure

All interviews were conducted by NRR, a master’s student at the time. Semi-structured interviews were conducted using an interview topic guide (Supplementary Material 2). The topic guide was reviewed by clinical professionals within the study team and pilot tested by NR and PP. Only participants and the researcher was present for all interviews. To establish rapport interviews were semi-structured, which provided the opportunity to commence interviews by initially discussing participants’ professional backgrounds and interest in the area of study during informal introductions prior to asking more specific topic related questions. For all interviews conducted over Microsoft Teams NRR kept her camera on. enabling an interaction that was close to face-face with the potential to develop rapport. Interviews were conducted once and not repeated. The guide explored topics such as clinicians’ professional backgrounds, how clinicians approached supporting people from ethnic minority groups and how confident they felt in doing so. Furthermore, clinicians were also asked about differences when interacting with patients from White British and ethnic minority groups, barriers that might exist to seeking mental health support, how to overcome these barriers and accommodating patients. Probes were used to further explore clinician views.

Interviews were carried out between February and April 2023. Thirteen interviews were conducted via Microsoft Teams, while one took place over telephone, lasting between 25 and 90 min. Interviews were recorded using the built-in recording and transcription software used to capture interview data concurrently on Microsoft Teams and using a Dictaphone for the telephone interview. Clinicians were assigned unique participant numbers to preserve anonymity, with these participant numbers being attached to their respective recordings and transcripts. Field notes were also made directly after interviews and attached to transcripts. No participants withdrew or refused to participate in interviews. Transcripts were not returned to participants following data collection for further comments and findings were not shared prior to publication. The research team reached this decision based on the expectation that clinicians would not have capacity to review manuscripts or findings alongside their workload and were satisfied with the researchers listening to interview recordings to correct transcripts. Data was analysed using reflexive thematic analysis (RTA), according to the six-step process outlined by Braun and Clarke (2021). RTA was selected as the focus of the research was to identify commonalities and differences between healthcare practitioners’ experiences by taking an exploratory approach, experience-driven rather than theory-led to aid understanding of barriers for patients from ethnic minority backgrounds. RTA is flexible and widely used to analyse qualitative data, involving reflexive engagement with the dataset [38]. The first step (See Table 1) involved NRR becoming immersed in the data while conducting the interviews and transcribing the data. Transcripts were read several times to make sense of the data and notes on analytical observations and initial feelings and thoughts were made. This contributed to reflexivity, an integral part of the analysis. The second stage involved developing initial codes and patterns of meaning in the data, this was done using NVivo 14. An inductive, data driven approach to data coding was adopted and codes were not pre-determined. Codes were discussed with SP, PP and CH and refined to enhance transparency around analytic rigor. To generate initial themes, codes with a shared meaning were grouped to create themes. Themes were developed and re-reviewed in relation to the research question. This stage involved going back and forth between the data to develop the narrative and reconsulting transcripts when needed.

20% of interview transcripts were also coded by SP and CLH. Codes and themes were subsequently discussed and refined as a research team. Stages 4 and 5 involved re-reviewing the codes and themes until themes became stabilised and the research team reached agreement. The final stage involved writing up the themes and sub-themes to develop a narrative of clinicians’ experiences of supporting patients from ethnic minority groups.

Reflexive analysis allowed for the interpretation of data based on personal knowledge and beliefs [38]. NRR had previous experience in qualitative interviewing during postgraduate study and recognised her positionality as someone from an ethnic minority background. Strategies to enhance reflexivity and minimise bias included making notes on initial analytical observations and insights and discussing these at length with the research team, keeping a reflective diary of assumptions, biases and subjective knowledge.

**Table 1:** Table presenting six-stage reflexive thematic analysis process

Stage		Research team members involved	RTA Process
1	Data immersion during interviews and production of transcripts	NRR, PP	• Transcripts finalised and reread to understand data • Notes on initial observations, thoughts and feelings made
2	Initial coding of transcripts	NRR, SH, PP, CLH	• Codes developed through recognised patterns within data to begin identifying common themes across transcripts
3	Codes with shared meaning grouped	NRR, SH, PP, CLH	• Initial themes generated by grouping relevant codes • Emerging subthemes identified
4	Theme development	NRR, SH, PP, CLH	• Themes and subthemes reviewed and reorganised based on relevance to research questions • Data within and between transcripts reconsulted to maintain fidelity to interviews and research questions
5	Finalisation of themes	NRR, SH, PP, CLH	• Themes and subthemes reviewed for final time and named • Themes and subthemes organised to begin report production
6	Report writing	NRR, SH, PP, CLH	• Identified themes and subthemes used to construct narrative of interpretation of data

## Results

A total of 14 clinicians were interviewed. Table 2 provides an overview of the demographics of participants; ethnic background was presented as directly stated by participants. A majority of the sample were mental health professionals (9/14; 64.3%), of varying professions, and five (35.7%) were General Practitioners.

**Table 2:** Table presenting sociodemographic characteristics of participants

Participant sociodemographic characteristics	Clinicians (*N* = 14)
	*n* (%)
Gender	
Male	11 (78.6)
**Ethnicity**	
White British	8 (57.1)
Asian British	2 (14.3)
Mixed	2 (14.3)
African	1 (7.1)
White Other	1 (7.1)
**Clinician role**	
General Practitioner	5 (35.7)
Clinical Psychologist	3 (21.4)
Trainee Psychiatrist/Psychiatrist	3 (21.4)
Psychotherapist	2 (14.3)
Mental Health Practitioner*	1 (7.1)
**Years of experience**	
Mean (SD)	14.1 years (10.3)
Range	2.5–37 years
**Geographical location**	
Nottinghamshire	10 (71.4)
Leicester	1 (7.1)
Oxfordshire	1 (7.1)
Gloucester	1 (7.1)
Birmingham	1 (7.1)

Three main themes and nine subthemes were identified (see Table 3). The three main themes were:

1. Clinician personal factors impacting their approaches to supporting patients from ethnic minority groups.
2. Clinicians’ perspectives on how patients perceive mental health and mental health services.
3. Structural factors and level of training provided affects consultation and treatment.

**Table 3:** Table illustrating themes and subthemes identified through reflexive thematic analysis

Main Themes	Subthemes
1. Clinician personal factors impacting their approaches to supporting patients from ethnic minority groups	1a) The role of effective clinician-patient communication in caring for individuals from ethnic minority groups.
	1b) Clinicians adapt their consultation and treatment approach based on perceived need
2. Clinicians’ perspectives on how patients perceive mental health and mental health services	2a) Recognition of mental health and the associated stigma within ethnic minority groups
	2b) Frustrations and mistrust with the Western mental healthcare system.
	2c) The impact of clinician ethnicity on the clinician–patient relationship
3. Structural factors and level of training provided affects consultation and treatment	3a) The impact of service pressures, time, and cost constraints on consultations and treatment
	3b) Enhancing training opportunities to better support patients from ethnic minority backgrounds
	3c) The importance of providing diverse referral options for accessing services
	3d) The need to enhance the information shared with patients and promote a diverse workforce

### Theme 1: clinician personal factors impacting their approaches to supporting patients from ethnic minority groups

The first theme centered on personal factors related to clinicians’ experiences in delivering support and the impact this had on the mental healthcare process. It highlighted the diverse communication strategies clinicians used when working with patients from ethnic minority backgrounds, including how they communicated information, the language choices made, and adjustments in consultation methods tailored to individual needs.

#### 1a) The role of effective clinician-patient communciation in caring for individuals from ethnic minority groups

Clinicians frequently expressed that when communicating with services users, they adapted their interactional approach depending on patient needs. Many clinicians recognised the importance of the language used when supporting patients from ethnic minority backgrounds and acknowledged that Western mental health terminology may be unfamiliar to these groups. Adaptations included a consideration of the terminology clinicians used when discussing mental health and avoiding medicalisation of terms as explained below.

*What I’ve tried to learn from that experience where it went wrong was to stick close to the client’s words*,* so it might still be thinking that they’ve got anxiety or depression*,* but if they’re saying ‘low mood*,*’ to try and stick with ‘low mood*,*’ and if they’re saying ‘unhappy*,*’ I’ll try to stick with that and not bring in that medical terminology*,* so trying to stay closer to the client’s frame of reference*. (P06, psychotherapist, White British ethnic background)

Moreover, clinicians also discussed making additional time to explain mental health-related terminology to patients, allowing them to better understand their symptoms. They suggested that this might be beneficial as Western ideas and open discussions around mental health may not be common within ethnic minority communities.

*I think some of the terminology needs further explaining*,* because this is kind of a new world really*,* for many patients.* […] *Seemingly people from ethnic minorities very rarely have similar conversations*. (P04, mental health practitioner, White British ethnic background)

Some clinicians also adopted terms from patients’ native language, as these terms facilitated communication by structuring explanations within patients’ personal understanding.

*I think that’s why I was using Bengali words*,* because they kept coming up. It made a lot more sense to that family in terms of describing their experiences*,* phenomena*,* patterns*,* that kind of stuff.* (P05, clinical psychologist, White Other ethnic background).

#### 1b) Clinicians adapt their consultation and treatment approach based on perceived need

Clinicians stated that their treatment approach depended on individuals’ experiences in relation to their mental health difficulties and that they did not always adapt their approach based on an individual’s ethnicity.

*I personally wouldn’t take a different approach just because they were from an ethnic minority group*,* I think. […] As long as you are trying to understand the relevant factors that are influencing somebody’s mental health*,* […] sometimes*,* their ethnicity is relevant to the formulation you put together.* (P03, clinical psychologist)

*I wouldn’t treat them as any different to anyone else*,* really. You have to*,* as with any person you have*,* take them as an individual and understand their cultural differences*,* […] there’s no stereotyping.* (P10, GP, Mixed ethnic background)

Clinicians also highlighted the importance of taking the time to build an alliance with patients from ethnic minority groups:

*In all the families I’ve worked with*,* it has taken time*,* a long time*,* longer than it probably has taken with clients from White British backgrounds and you’ve got to be pretty persistent.* (P12, psychotherapist, White British ethnic background)

Some clinicians also noted that they incorporated patients’ religious beliefs to help them manage their distress, to improve engagement with support.

*[…] Some clients who had migrated from Africa. I think it might have been Somalia*,* Sudan*,* but it was a Coptic religion*,* so they had there was a lot of kind of religious beliefs that came into that*,* but we used them as resources*,* you know*,* these particular beliefs are a resource in getting you through this eating disorder problem.* (P05, clinical psychologist, White Other ethnic background)

To address language barriers, clinicians discussed the involvement of interpreters during GP appointments or therapy sessions to facilitate communication.

*If I’ve got to a stage where I think I need an interpreter*,* I often personally often find it very helpful and I think patients also find it helpful*,* because you can get a better depth of understanding of what’s going on. (P02*,* GP*,* White British ethnic background)*

However, it was also noted that this could introduce additional dynamics into sessions, as interpreters might project their own views onto patients’ experiences, potentially diminishing the authenticity of a patient’s personal accounts of their difficulties.

*It really does depend on the interpreter because they don’t always translate exactly what the client’s saying. They’re just rephrasing things*,* but also lots of things are lost. (P05*,* clinical psychologist*,* White Other ethnic background)*

### Theme 2: clinicians’ perspectives on how patients perceive mental health and mental health services

Clinicians’ perspectives on how patients from ethnic minority groups viewed mental health and related services reflected complex and sometimes conflicting insights. Clinicians conveyed that patient perspectives were shaped by a range of factors, including cultural norms, perceptions about Western healthcare systems and the potential influence of the clinician’s ethnicity on the clinician-patient relationship.

#### 2a) Recognition of mental health and the associated stigma within ethnic minority groups

Clinicians frequently noted that patients associated their mental health difficulties with religious or spiritual beliefs. Furthermore, they observed that patients often reported physical symptoms that were indicative of underlying mental health issues, indicating that some individuals from ethnic minority backgrounds might somatise their symptoms.

*People from Asian backgrounds might actually present with often what looked like physical sort of problems*,* maybe fatigue or somatic complaints. […] We might say*,* ‘that’s depression*,*’ but actually what they’re saying is ‘I’m tired.’ So I think probably there are kind of cultural differences in what people identify as being the problem for them*. (P03, clinical psychologist, Asian British ethnic background).

Clinicians frequently discussed the effect of stigma surrounding mental health difficulties on patients. Stigma related to mental health appeared to be multi-faceted, evoking different reactions but impacting patients’ engagement with mental healthcare in similar ways. Clinicians described mental health difficulties to be primarily perceived negatively by patients and their families. For example, experiencing poor mental health was seen as being humiliating. This may have elicited fear of being regarded negatively by other members of their community.

*You’re not getting up out of bed or you’re not getting washed. Within these faith communities*,* that’s a huge embarrassment for those families*. (P12, psychotherapist, White British ethnic background).

When asked about barriers to seeking mental health support, a mental health practitioner suggested that a barrier for international students was fear of information being shared with their parents. This seemed to be attributed to embarrassment about experiencing mental health difficulties.

*That they believe that parents will be informed*,* even if they’re on the other side of the world.* (P04, mental health practitioner, White British ethnic background).

Similarly, other clinicians commented on these negative perceptions and differing attributions around mental health. They were held not only by patients, but also their families, and influenced the acceptance of treatment or seeking support for mental health difficulties.

*Sometimes patients from ethnic backgrounds are less prepared in terms of knowing what services are out there*,* sometimes due to cultural reasons that they’ve been kind of*,* in their words*,* discouraged by parents or older siblings.* (P04, mental health practitioner, White British ethnic background).

Furthermore, struggling with mental health could also be perceived as a sign of failure, which may have reduced patients’ confidence and willingness to engage with treatment.

*I guess*,* in particular*,* what I’ve experienced of it almost being like a ‘personal failing’ kind of thing. So that kind of has made it harder for us to get into stuff or made it harder for them to make changes*. (P08, clinical psychologist, Mixed ethnic background).

Overall, this suggested that stigma was rooted within ethnic minority groups overall, extending beyond patients to their families and other members of their community and seemed to be a major barrier to accessing mental health support.

#### 2b) Frustrations and mistrust with the Western mental healthcare system

Clinicians acknowledged that differences between healthcare systems could discourage patients from seeking help, largely due to their unfamiliarity with Western practices. Clinicians expressed that cultural background, and beliefs could influence how treatment for mental health was sought. Some clinicians noted that many non-Western healthcare systems tend to prioritise pharmacological interventions over therapeutic approaches.

*Many cultures*,* it’s quite a more medicalised framework*,* you know*,* it needs medicines to sort it out and drugs*. (P02, GP, White British ethnic background).

Furthermore, some patients preferred traditional therapies over Western interventions. It seemed that differing treatment models and cultural dissonance around understanding mental health could induce discomfort in patients from ethnic minority backgrounds, evoking mistrust and reluctance to embrace a Western approach.

*You go to healers and you get stuff*,* which is not tested*,* but is traditional and the whole of Western medicine is rejected.* (P09, psychiatrist, White British ethnic background)

Indeed, clinicians observed that some patients from ethnic minority groups exhibited mistrust or skepticism towards mental health services. This mistrust often led to delays in seeking services until their symptoms became severe enough to necessitate inpatient care. Alternatively, this could be interpreted as patients exhibiting mistrust due to being sectioned under the Mental Health Act, potentially against their will.

*The most difficult group to engage and really*,* I think it really requires a different approach*,* is the Black and Afro-Caribbean group*,* because they just do not trust us.* […] *You see them late and then usually they’ve been detained under the Mental Health Act or in inpatient and they’re mistrustful*. (P09, psychiatrist, White British ethnic background).

This might have also been related to experiences of racism within the healthcare system, which may have increased patients’ distrust in clinicians and services when receiving mental health support. Clinicians suggested that some patients may have experienced poor treatment within mental health services, due to racial discrimination. However, this seemed to extend beyond healthcare services and was described by clinicians to be a broader, systemic issue, contributing to patients’ mistrust in systems overall.

*I think there probably is a lot of racism to be honest*,* both in terms of how they’re treated by the system and more broadly*.*[…] I think staff can often be a bit racist as well*,* in hospitals and community mental health teams. […] I suppose it’s a bit of overlap there*,* isn’t there*,* but I think there can often be a distrust in Western healthcare institutions and particularly institutions in general. Often we work closely with the police as well*,* so obviously that makes it more difficult. And often we are reliant*,* we kind of need to share information as well with social care and with the government and with the police. So I think*,* understandably*,* that raises a lot of worries and anxieties for people* (P07, psychiatrist, White British ethnic background).

Mistrust also appeared to stem from the belief that clinicians would not be able to understand or relate to the patient’s perception of their challenges, which in turn could reduce engagement and openness to accessing healthcare services.

*They may also just not want to engage because they don’t believe the system would support- trust as a system is a problem for them and they don’t believe the person that is going to be there is going to understand their problems*,* so why bother?* (P01, GP, African ethnic background).

Many clinicians expressed that mental health support is not a “one-size-fits-all” approach. They noted recent shifts in mental health services and among clinicians, greater openness in incorporating non-Western perspectives on mental health. This included patients, developing their own understanding of their mental health difficulties. It seemed that evolving approaches to clinical practice, including assimilating patients’ own understanding of their mental health difficulties within interventions, could be utilised as a mechanism to overcome mistrust and negative outlooks on Western mental healthcare.

*It’s a shifting culture* […] *because usually people would be told and labelled as ‘you lack insight’ if you didn’t acknowledge that you had this diagnosis. You’re trying to slide*,* pass it off as a spiritual experience. Now there seems to be less of an onus on somebody accepting their diagnosis and more acceptance that it’s about you finding an understanding and a path*. (P12, psychotherapist, White British ethnic background).

#### 2c) The impact of clinician ethnicity on the clinician–patient relationship

Interviews with clinicians highlighted that in some instances, patients from ethnic minority groups preferred to consult with a clinician from a similar ethnic minority background, as they felt they would be better understood.

*I do remember specific cases where*,* perhaps because I’m from a South Asian ethnic background*,* other people would come to see me and perhaps feel that I understand them better or even without saying it*,* they would feel that we had some sort of an understanding.* (P13, GP, Asian British ethnic background).

When clinicians were asked about how they felt that their own ethnic background affected their interactions with patients from ethnic minority backgrounds, they gave two common, polarised responses. Some clinicians seemed less aware of how their own ethnicity impacted their support of patients, whereas for others this was more pertinent.

*I’m not sure. I’m sure there is an impact. Do patients from ethnic minority groups prefer to see somebody from the same ethnic minority group? I guess they do*,* I don’t know.* (P11, GP, White British ethnic background).

Conversely, another clinician recognised that their ethnic background made them less familiar with patients’ experiences of mental health challenges and local demand within their service.

*…perhaps because of my background*,* definitely there’s a gap between kind of what some of my service leads probably would benefit from or need and then what I’m necessarily able to provide.* (P14, trainee psychiatrist, White British ethnic background).

Clinicians also mentioned certain challenges of being from an ethnic minority background when interacting with patients, noting that professional boundaries could sometimes become obscure. One GP described needing to take on the role of both cultural advocate and doctor:

*I don’t want to be put in that situation*,* because then the boundaries get blurred between who’s the doctor and who’s the interpreter and who’s the cultural advocate*. (P13, GP, Asian British ethnic background).

### Theme 3: structural factors and level of training provided affects consultation and treatment

The final theme highlighted that structural factors, including service pressures, high demands and inadequate training, could adversely impact the efficacy of support provided to patients from ethnic minority groups. Clinicians often shared suggested improvements in relation to these barriers. This included offering additional training, establishing multiple referral pathways, promoting diversity within the mental healthcare workforce and improving health information for patients to increase accessibility and engagement with mental health services for ethnic minority communities.

#### 3a) The impact of service pressures, time, and cost constraints on consultations and treatment

Clinicians highlighted clinical pressures and time limitations affecting their capacity to effectively support patients from ethnic minority backgrounds. The NHS was frequently described as an overburdened service, which had a direct impact on the quality of consultations.

*The NHS is working under so much pressure*,* so it’s easier to get patients in and out quickly*,* like everybody else*,* rather than having tailor-made appointment slots.* (P01, GP, African ethnic background).

A psychotherapist described finding long waiting lists and the pressure from management to achieve positive outcomes to be stressors when formerly working within IAPT services. This pressure was compounded by the expectation that recovery rates among patients from ethnic minority groups would be lower, which could reflect poorly on clinician performance.

*Your scrutiny on your therapeutic outcomes is intense and there’s a fear of sanction or reprimand if you get a few bad cases together. […] Then you get this person from an overtly kind of different cultural background. I used to experience this little nagging doubt like*,* oh no*,* another person I’m not really going to be able to help. And then this is going to actually blow back on me too.* (P06, psychotherapist, White British ethnic background).

High demand placed additional strain on services, stretching both time and financial resources. Consequently, accommodations like having interpreters present seemed less feasible.

*Generally*,* when the interpreter is there*,* it often goes quite well. […] But it’s quite expensive and often people don’t want to do it and takes a lot more time.* (P07, psychiatrist, White British ethnic background).

These resource limitations also contributed to high thresholds for admission and referral acceptance into mental health services, ensuring that available resources were directed toward patients with more complex needs. However, this approach often resulted in those with less severe difficulties, as well as individuals needing additional accommodations, being overlooked by the system.

*Under normal circumstances*,* the healthcare pathways are not responsive to unique needs*,* like being from ethnic minorities. The healthcare pathways and mental health are designed to be difficult to access and in order to protect the resources*,* patients have to be very unwell before they are accepted.* (P13, GP, Asian British ethnic background).

Thus, while clinicians acknowledged both personal and challenges perceived by patients in providing effective support to individuals from ethnic minority backgrounds, systemic barriers within the service structure, that added further obstacles were also identified.

When discussing these broader systemic issues, a clinician stated that the priority should instead be on cultivating resilience within communities. Rather than pathologising hardships that people from ethnic minority groups face. They suggested that a preventative approach, wherein communities are equipped with the resources to prevent hardships that may lead to mental health difficulties, may be more cost-effective and beneficial in the long term. Additionally, they suggested that fortifying communities may improve their engagement and relations with NHS mental health services.

*So yes*,* there’s all the cultural sensitivity*,* looking at access. But actually it’s much more interested in that sort of upstream of how do we make our communities resilient? How do we support people so they don’t need to medicalise difficult lives? […] Rather than going to communities*,* you know*,* ‘looks like there’s an issue*,* what help do you need to sort that problem yourself*,* rather than saying oh*,* it’s a problem*,* we’ll put a service in for you*,*’ and the more you do that*,* the more you weaken the community. So it becomes a self-fulfilling expensive thing. If you can support vibrant*,* healthy communities and that are well connected*,* that actually flow into the NHS*,* it becomes much less* [costly]. (P02, GP, White British ethnic background).

#### 3b) Enhancing training opportunities to better support patients from ethnic minority backgrounds

In relation to these service constraints, clinicians commonly referred to training. This included training they had already received but also detailed various proposed improvements to training. Clinicians within both primary and secondary care had received differing levels of training that was oriented towards supporting patients from ethnic minority groups.

*When I was training and this was a long time ago*,* 2010*,* there was no training on adapting CBT to fit with people from different cultural backgrounds*. (P06, psychotherapist, White British ethnic background).

During discussions about factors that contributed to clinicians’ confidence in supporting patients from ethnic minority groups, the importance of receiving culturally sensitive, tailored training was emphasised. This seemed to primarily be related to helping clinicians feel more equipped to support patients from ethnic minority backgrounds and more confident in considering culture when delivering mental healthcare. Other suggested clinician training around supporting patients from diverse backgrounds was mentioned including making accommodations that may be particularly relevant to ethnic minority groups. This included cultural awareness and working with interpreters and was also perceived to be useful.

*I think having probably*,* having training for some NHS departments and clinicians would be really good. Training in using interpreters*,* training around just cultural sensitivity*. (P03, clinical psychologist, Asian British ethnic background).

Additionally, alongside culturally sensitive training, some clinicians mentioned that based on local populations and mental healthcare needs, culturally specific training may be more useful.

*‘Culture specific is if you had a huge population say from the Indian subcontinent*,* you may want to service that’s*,* sort of*,* specific for that sort of group. […] And there’s some advantages*,* […] but it’s not viable option at all times. I always say you tend to go to the more sort of*,* try and make them as culturally sensitive as you can. Occasionally with important areas of a large population*,* you might do something kind of culturally specific.’* (P02, GP, White British ethnic background).

More extensively, a clinician emphasised that training around improving therapeutic skill overall would help clinicians support patients from any background. This seemed to be more related to making mental health support more personalised over culturally relevant.

*I do think there’s a piece of this which is cultural competence*,* but I actually think*,* if you really sharpen your therapeutic skills*,* it’s likely that you’re going to be able to help people from a diverse range of backgrounds. […] I think there are actually some specific skills about making things more personally relevant*,* meaningful to anyone*,* which is like a broader therapeutic skill*. (P08, clinical psychologist, Mixed ethnic background).

Furthermore, training was recommended for clinicians from ethnic minority groups to support interactions with patients of the same ethnic background, particularly in managing cultural and religious beliefs.

*There’s possibly even a case for specific training for people from ethnic minority groups who are treating other patients from their own ethnic group and how to deal with religious and cultural beliefs*. (P13, GP, Asian British ethnic background).

Clinicians also commented on the need to carry out more research on how to make clinician training more beneficial:

*Yeah*,* I think that’s really important. Like I said*,* all the research has shown at the moment is there’s a massive gap difference and disparity. So I think a lot more work needs to be done on*,* one*,* understanding why that is and then*,* two*,* what we can do as individuals and services to not just reduce the numbers*,* but also make people’s experiences a much more positive one*. (P14, trainee psychiatrist, White British ethnic background).

*The two things that I’ve mentioned are really important to me*,* actually supporting therapists to get better at their clinical skills. There aren’t really many studies* [on this]. (P08, clinical psychologist, Mixed ethnic background).

#### 3c) The importance of providing diverse referral options for accessing services

The need for improvement to accessibility of mental healthcare for ethnic minority groups and how services could be transformed was also discussed. Some clinicians highlighted the benefits of providing multiple access options, such as online appointments, to improve patients’ ability to engage with services. This approach was seen as particularly valuable in addressing stigma, which could make it difficult for individuals from ethnic minority groups to seek mental health support openly.

*Some people within* [ethnic minority] *groups prefer to have digital communication*,* like this*,* because they can just escape into a backroom.* (P09, psychiatrist, White British ethnic background).

Interviews suggested that community-based approaches could be a potential intervention method to engage with ethnic minority communities. Outreach using community-based approaches could be beneficial, as patients might feel more comfortable in familiar environments outside traditional healthcare settings.

*I’m thinking of a couple of psychologists I know who do outreach clinics and things like local libraries. So*,* completely non-stigmatising environments.* […] *They have a confidential room in the back of the library*,* people can go and see them there and that’s a really good idea. *(P06, psychotherapist, White British ethnic background).

However, another clinician expressed concerns about implementing community-based approaches in religious settings. They believed that religious leaders might struggle to incorporate Western therapeutic concepts related to mental health into their existing frameworks and practices. Therefore, it may be difficult to locate a midpoint when developing a transcultural approach, in which both Western and traditional approaches were integrated, while preserving fundamental ownership and cultural values.

*My experience of religious people is that their own religious beliefs are so deep-set and strong*,* that it’s unlikely that they’re going to completely transform the way that they practise and preach and I’d be reluctant for the NHS to start using community leaders and religious people as their avenue for public health interventions*.’ (P13, GP, Asian British ethnic background).

Therefore, while community-based approaches might have some advantages, in some contexts they may be less plausible, as community members might feel less inclined to support these interventions.

Additionally, another clinician stressed the importance of adopting a preventative, collaborative approach to support. They suggested that individuals could be evaluated for signs of mental health difficulties and clinicians could collaborate with patients based on their needs and understanding, ultimately determining the best ways to provide support.

*So*,* do routine*,* kind of*,* outcome assessments*,* routine assessment of screening of psychological difficulties*,* but then go collaborative in the way that you feedback those results.* (P08, clinical psychologist, Mixed ethnic background).

In this way, this might allow patients from ethnic minority groups to feel more control when receiving mental health support, particularly in contexts where they feel distrusting of healthcare professionals and less familiar with Western healthcare practices and systems.

#### 3d) The need to enhance the information shared with patients and promote a diverse workforce

Clinicians also highlighted that the information provided to patients, particularly regarding mental and emotional distress, needed to be improved so that there was greater clarity about the mental health services offered. Clinicians noted that patients who were better informed about Western norms and mental health services were often more willing to seek support.

*I think for me it’s less about age*,* maybe more around* [Western] *acculturation or understanding what the services can offer.* (P06, psychotherapist, White British ethnic background).

*So often it really is education*,* it’s awareness*,* recognition and knowing how to*,* you know*,* I guess navigate the healthcare system. So*,* you can seek the appropriate support at the right time. You know*,* for example*,* you don’t always need to see a doctor for mental health. You can self-refer yourself to all these talking therapies.* (P10, GP, Mixed ethnic background).

That data also emphasised that any strategies to promote healthcare use should consider intra and inter group differences and not treat ethnic minority communities as a homogeneous, cohesive entity.

*I think increasingly we will need more research on specific ethnic groups*,* because I think grouping all ethnic minorities together is very challenging*. (P13, GP, Asian British ethnic background).

Additionally, it was also acknowledged that there needed to be more diversity within the workforce. When evaluating the NHS workforce, some clinicians expressed concerns about its lack of diversity and the need for more clinicians from ethnically diverse backgrounds. They emphasised that individuals from ethnic minority groups should occupy positions at all levels of the NHS, including senior professional roles, and that greater attention should be given to training a diverse workforce. Clinicians suggested that better representation may in turn increase identification with and trust in mental health services.

*To wait for a situation where 10*,* 20*,* 30 years down the line you have poor representation of people that look like a place you want to serve and only to make a token gesture of training people to come in*,* that in my opinion is not sufficient*. (P01, GP, African ethnic background).

*I think the make-up of mental support services*,* so um*,* IAPT services*,* I think you know often*,* if they can reflect the population that they serve*,* because trust is so important*,* especially with the therapist*,* then I think that that’s definitely beneficial.* (P10, GP, Mixed ethnic background).

## Discussion

This qualitative study explored clinicians’ experiences in supporting adults from ethnic minority groups with their mental health in England. The findings revealed that while clinicians generally felt confident in their ability to provide support, they described several barriers relating to delivering effective care, as well as perceived barriers for patients to access care. These could be broadly classified into patient, clinician, and organisational barriers. Patient barriers included language difficulties, stigma, varying levels of understanding about mental health, mistrust, and differences in mental healthcare practices. Clinician or organisational barriers comprised issues related to service pressure and resource limitations. Clinicians differed in how much they adapted their approaches based on patients’ ethnic backgrounds but often took cultural differences into account and collaborated with interpreters when needed. To enhance the support provided by mental health services for ethnic minority groups, clinicians recommended increased research and training, improved information for patients about NHS referral pathways and services and adjustments in how mental health support is delivered to communities.

The present findings are consistent with previous research highlighting that limited mental health visibility, language barriers, stigma, discrimination and clinicians lacking cultural awareness discourage patients from ethnic minority groups from accessing support [22]. Reinforcing the findings of Faheem (2023), clinicians in the current study reported being less able to consider other cultures due to lack of knowledge [19]. Furthermore, clinicians were also concerned that treatment progress and rapport could be impeded by cultural differences. Clinicians felt that patients might favour receiving support from clinicians from a similar ethnic background and extended this by suggesting that the NHS mental healthcare workforce should be more diverse at senior and patient-facing levels. The need for cultural adaptation, where appropriate, and the need for more training was also recognised by clinicians.

In line with previous research [17], many clinicians felt that mental health services lacked cultural sensitivity, as care frameworks and evidence-based practice were based on Eurocentric research.

Our findings also revealed that clinicians would welcome better training around cultural competency when engaging patients. All clinical psychologists in the present study had received such training, with at least one commenting that their training had aided their ability to be receptive to cultural factors. Meanwhile, a majority of GPs had received training that was often described to be early on during medical school. Despite less training, all GPs stated that they felt confident when supporting ethnic minority communities, mostly attributing this to clinical experience. These findings support a previous literature review which found that most cultural competency training offered to GPs was not adequately developed or regulated, with GPs instead developing their competency through active practice [39]. Therefore, this suggest that improved, up-to-date cultural competency training could be beneficial for healthcare professionals. This training could assist them in considering patients’ cultural and personal understanding of mental health and the imapct of their own ethnicty during the care process. However, when comparing training in primary and secondary care services, while differences were identified, the evidence is insufficient to draw strong comparative conclusions. Given that the scope of this research did not allow for in-depth exploration of the nature of cultural competence training currently received by clinicians, we are less suited to put forth recommendations for training. Instead, we propose that current training requires review and regulation as an initial step. Indeed, further research is needed to understand how cultural competence is implemented within GP training and on how to improve current cultural competence training available for mental health professionals in England [32].

It was also recognised by clinicians that patients might show a preference towards clinicians from a similar ethnic background, due to shared characteristics and cultural understanding. Within mental health treatment, ethnic matching, can be a considered to be a positive form of cultural adaptation [28]. However, as highlighted by some clinicians within the study, most professional clinical positions within the NHS were held by people from a White British background [40], making ethnic matching challenging. Therefore, clinicians proposed that better representation within the mental health workforce might improve patients’ attitudes towards and engagement with treatment. However, this might not translate to treatment success. A meta-analysis of studies on ethnic matching found that although clients favoured therapists from the same ethnic background, ethnic matching did not improve treatment outcomes [29]. However, clients did perceive therapists from the same ethnic background more positively, suggesting that this form of adaptation may still improve engagement and openness to mental health treatment.

Clinicians discussed services being restricted by funding and time and that this affected their ability to provide adequate support generally, but also specifically to patients from ethnic minority backgrounds. Based on this resource strain and ethnic minority groups being less likely to seek mental health support, alternative modes of delivering support should be considered. Clinicians proposed that community-centred methods could be useful in terms of reaching people in need of support and that establishing preventative structures and resources could build resilience, which may potentially serve as a protective factor against mental health difficulties. Furthermore, existing research suggests that previous mistreatment that was believed to be racially-motivated contributes to mistrust of mental health services [23]. Developing community-based interventions in environments that people from ethnic minority backgrounds feel safe in could potentially rebuild their trust in mental healthcare and their willingness to engage. They may also reduce mental health stigma within communities [21]. Additionally, previous research has indicated that ethnic minority groups may be more likely to present later to services, when mental health difficulties are more severe [17]. Fostering community-led outreach services may support with providing early or preventative intervention. Based on current findings, these outreach services should not focus on forcing communities to adopt certain beliefs or practices but instead focus on building knowledge and awareness about NHS mental health services and building resilience.

### Strengths and limitations

This qualitative study provided an important insight into the experiences of clinicians working with members of ethnic minority communities with mental health difficulties. Whilst the findings are not intended to be generalisable across all mental healthcare providers in England, they offer contextually grounded insights into clinicians’ experiences, which may be transferable to similar settings. This study was strengthened by the inclusion of clinicians from a variety of professional backgrounds, such as GPs, clinical psychologists, psychiatrists and psychotherapists. This meant that a range of experiences were able to be explored, including those within primary and secondary care. The semi-structured interview schedule allowed for flexibility in topic discussion, whilst supporting rich discussion of topics.

A qualitative approach allowed for the interpretation of interlinked factors from various levels, including patient, clinician and organisational factors. Connections between these factors were tangible, allowing the research team to interpret clinicians’ accounts of supporting patients from ethnic minority groups. The researcher conducting the interviews and leading the analysis was from an ethnic minority background. They had personally witnessed cultural resistance to seeking mental health support in people from ethnic minority groups, as well as poor treatment of patients. These experiences and the researcher’s ethnicity carried into this study and may have both positively and negatively affected data collection and interpretation. Offering participants the opportunity to review transcripts would have enabled member-checking, a valuable strategy to enhance credibility of the findings.

A snowballing technique had to be applied to recruit participants as utilising a convenience sampling approach did not yield many participants. This meant that most of the participants were known to the study team. The present findings may therefore be prone to selection bias and those who felt more comfortable sharing their thoughts and willing to participate. This may have impacted on how open participants felt about their experiences. However, the researcher conducting and analysing the interviews was not known to any participant, and as such, it is unlikely that this unduly resulted in any increased risk of bias, or concerns with power dynamics or anonymity. Disseminating study information more widely on social media may have enabled a diverse group of participants to be recruited, however resource constraints such as time did not allow for other recruitment channels to be utilised. An additional limitation was that interviews were only offered to be conducted remotely. This may have influenced participant disclosure as they may have felt more comfortable speaking in person. However, given that only one interview was conducted over telephone and the diverse views offered by the respondents, it can be inferred that participants voiced their thoughts openly and honestly.

A further limitation of the study was that the clinician sample was mainly White British and male. While some clinicians from ethnic minority groups were recruited, this was limited to mainly Asian, Black and African-Caribbean backgrounds. Although this is broadly representative of the composition of the NHS workforce [40] it is possible that clinicians belonging to ethnic minority groups may have different experiences to White British clinicians. The lack of diversity in the sample precluded any meaningful comparisons between White clinicians and clinicians from ethnic minority background, a limitation that has been previously reported in other similar studies [18]. Furthermore, ten out of fourteen participants were from the Nottinghamshire area which reflects the pragmatic and relational realities of qualitative recruitment, our small sample size may have impacted theme saturation and depth. Representativeness and geographical distribution are critical considerations in qualitative research, particularly given the influence of differing social and environmental contexts on participants’ experiences. In this study, the geographical distribution of participants was skewed toward Nottinghamshire primarily due to accessibility and time limitations. This concentration may limit the extent to which the findings can be extrapolated to the entire study area, the data nonetheless provide in-depth insights into the lived experiences within this specific context. Importantly, the inclusion of four participants from other regions strengthened the study by introducing variation in perspectives and enabling broader reflection on shared and divergent experiences across settings. These findings should however be interpreted with caution, as reliance on data from a limited geographical segment may introduce contextual bias. Future studies should aim to include a broader geographical representation to capture potential variations across different social environments and strengthen the transferability of the findings. We were also unable to explore intersectionality, aspects such as gender and religion may have also influenced participant views and warrants further research Future studies should explore how intersectionality within ethnic minority groups and other patient factors, such as gender, age, socioeconomic status, and immigration status may interact with ethnicity to influence experiences and use of mental health services. Differences between ethnic minority groups should be explored, in order to identify cultural intricacies and differences in experiences of mental healthcare. Furthermore, in this study we did not seek the perspectives of managers or key decision makers. Future research should explore perspectives within different levels of service organisations, such as those in senior roles who have more authority to make the proposed changes to how mental health services are structured.

### Implications for practice

Enhancing cultural competency among clinicians may help reduce health inequalities. The findings from this research support the idea that current cultural competence training for mental health professionals should be reviewed. Further research is needed on how to improve cultural competence training available for mental health professionals in England. As previously mentioned, clinicians might benefit from enhanced cultural competence training and an awareness of how to adopt culturally sensitive and inclusive communication strategies. Here, the focus should be on cultural humility, fostering communication approaches that acknowledge and respect individual values and beliefs and encourage learning about them, alongside an awareness of one’s own cultural biases and views. Although interpreters may go some way to overcome language barriers, the focus should be broader, on a wider communication approach that facilitates trust-building and mitigates fears around stigma and confidentiality concerns. Ideally, interpreters should be trained to understand medical and mental health terminology to facilitate effective communication. Public health campaigns, developed and delivered alongside community leaders or trusted figures within ethnic minority communities might improve awareness and acceptability of mental health services. Such campaigns might focus on reducing stigma associated with mental health or informing communities about the availability and importance of seeking mental healthcare.

Further research is warranted on how mental health interventions might be adapted or personalised to align with the cultural norms, beliefs, and values of different groups to enhance engagement and outcomes. However, we acknowledge that these solutions will be accompanied by associated costs. These costs warrant increased priority from both researchers and policy makers to develop and evaluate support programs specifically designed to engage ethnic minority groups and test the clinical and cost-effectiveness of delivering them. These streams of work should be co-produced by people from ethnic minority groups with lived-experience of mental health difficulties and clinicians supporting these groups, with on-going consultation policy and decision makers.

## Conclusions

This research found barriers to care at patient, clinician, and organisation levels. These included barriers around language, stigma, mistrust of services, as well as service limitations and pressures. The findings underscore the need for a comprehensive, multi-level approach to addressing the barriers faced by ethnic minority groups in accessing mental health services. This includes equipping clinicians with the skills to provide culturally sensitive care, transforming systemic practices to be more inclusive and engaging communities to reduce stigma and improve trust in mental health services. These changes are paramount in supporting equitable mental health outcomes for all.

## Supplementary Information

Below is the link to the electronic supplementary material.

Supplementary Material 1

Supplementary Material 2

## Data Availability

The datasets generated and analysed during the current study are not publicly available due to confidentiality and privacy concerns because the data were produced from interviewing clinicians in England.  However, they are available from the corresponding author upon reasonable request.

## Abbreviations

GP: General Practitioner
IAPT: Improving Access to Psychological Therapies
NHS: National Health Service
NICE: National Institute for Health and Care Excellence
RTA: Reflexive Thematic Analysis
