# Supplementary material for: Navigating cultural barriers: a qualitative study exploring clinicians’ experiences of providing mental health support for ethnic minority groups in England
Source: BMC Health Serv Res. 2026 Feb 7;26:353. doi: 10.1186/s12913-026-14146-5 (PMC12977790; doi:10.1186/s12913-026-14146-5)
Supplement: Supplementary file 1 — Supplementary Material 1 [file 12913_2026_14146_MOESM1_ESM.pdf]

Consolidated criteria for reporting qualitative studies (COREQ): 32-item checklist

Developed from:
Tong A, Sainsbury P, Craig J. Consolidated criteria for reporting qualitative research (COREQ): a 32-item checklist for interviews and focus groups. *International Journal for Quality in Health Care*. 2007. Volume 19, Number 6: pp. 349 – 357

No. Item	Guide questions/description	Reported on Page #
**Domain 1: Research team and reflexivity**		
Personal Characteristics		
1. Interviewer/facilitator	Which author/s conducted the interview or focus group?	Page 12
2. Credentials	What were the researcher's credentials? E.g. PhD, MD	Page 12
3. Occupation	What was their occupation at the time of the study?	Page 12
4. Gender	Was the researcher male or female?	Page 12
5. Experience and training	What experience or training did the researcher have?	Page 13
Relationship with participants		
6. Relationship established	Was a relationship established prior to study commencement?	Page 12 .
7. Participant knowledge of the interviewer	What did the participants know about the researcher? e.g. personal goals, reasons for doing the research	Page 12
8. Interviewer characteristics	What characteristics were reported about the interviewer/facilitator? e.g. Bias, assumptions, reasons and interests in the research topic	Page 40

**Domain 2: study design**		
Theoretical framework		
9. Methodological orientation and Theory	What methodological orientation was stated to underpin the study? e.g. grounded theory, discourse analysis, ethnography, phenomenology, content analysis	Page 2 and 10
Participant selection		
10. Sampling	How were participants selected? e.g. purposive, convenience, consecutive, snowball	Page 11-12
11. Method of approach	How were participants approached? e.g. face-to-face, telephone, mail, email	Page 11-12
12. Sample size	How many participants were in the study?	Page 12
13. Non-participation	How many people refused to participate or dropped out? Reasons?	Page 11-12
Setting		
14. Setting of data collection	Where was the data collected? e.g. home, clinic, workplace	Page 12-13<br>.
15. Presence of non-participants	Was anyone else present besides the participants and researchers?	Page 13 Inferred as one to one interviews
16. Description of sample	What are the important characteristics of the sample? e.g. demographic data, date	Page 16
Data collection		
17. Interview guide	Were questions, prompts, guides provided by the authors? Was it pilot tested?	Additional file and page 12
18. Repeat interviews	Were repeat interviews carried out? If yes, how many?	No, inferred on page 12-13
19. Audio/visual recording	Did the research use audio or visual recording to collect the data?	Page 13

20. Field notes	Were field notes made during and/or after the interview or focus group?	Page 13
21. Duration	What was the duration of the interviews or focus group?	Page 13
22. Data saturation	Was data saturation discussed?	Page 12
23. Transcripts returned	Were transcripts returned to participants for comment and/or correction?	Page 13
**Domain 3: analysis and findings**		
Data analysis		
24. Number of data coders	How many data coders coded the data?	Page 14-15
25. Description of the coding tree	Did authors provide a description of the coding tree?	Page 14-15
26. Derivation of themes	Were themes identified in advance or derived from the data?	Page 11,13-15
27. Software	What software, if applicable, was used to manage the data?	Page 14
28. Participant checking	Did participants provide feedback on the findings?	Page 13
Reporting		
29. Quotations presented	Were participant quotations presented to illustrate the themes/findings? Was each quotation identified? e.g. participant number	Pages 18-36
30. Data and findings consistent	Was there consistency between the data presented and the findings?	Yes, there was. Page 18-36
31. Clarity of major themes	Were major themes clearly presented in the findings?	Yes. they were. From page 17-36
32. Clarity of minor themes	Is there a description of diverse cases or discussion of minor themes?	Discussion of major and minor themes From page 17-36
