# Supplementary material for: Navigating cultural barriers: a qualitative study exploring clinicians’ experiences of providing mental health support for ethnic minority groups in England
Source: BMC Health Serv Res. 2026 Feb 7;26:353. doi: 10.1186/s12913-026-14146-5 (PMC12977790; doi:10.1186/s12913-026-14146-5)
Supplement: Supplementary file 2 — Supplementary Material 2 [file 12913_2026_14146_MOESM2_ESM.pdf]

Supplementary Material 2: Interview topic guide

Part 1 – Introduction

- Interviewer introduction
- Explain why clinician was invited to participate

Participant information sheet

- *Voluntary participation*
- *Right to withdraw*
- *Recording of interview and confidentiality – anonymisation, start recording*
- *Questions*
- *Check consent form has been signed and reaffirm participant is happy to proceed*
- *Do you have an appointment/meeting after this, which leaves you restricted for time?*

Could you provide a brief summary of your professional background?

- *Job role/title*
- *Duration in role/practice as clinician*
- *Area of work*
- *Geographical region of work*

What are your experiences of supporting service users from ethnic minority groups with their mental health?

- *i.e., frequently or infrequently*

Part 2 – less experience with service users from ethnic minority groups

- What barriers do you think ethnic minority groups might face when seeking mental health support?

- How do these barriers differ from those for non-minority groups seeking mental health support?
- How might these barriers for ethnic minority groups be overcome?
- Is there anything that mental health services could work towards in order to better support service users from ethnic minority backgrounds?

Part 2 – prior experience with service users from ethnic minority groups

What characteristics did these service users have?

- *Nature of mental health difficulties*
- *Generation*
- *British-born*
- *Age*
- *Gender*

How confident do you feel in supporting service users from ethnic minority backgrounds?

- *Training*
- *Why do you feel confident?*
- *Why do you not feel confident?*

How did you approach discussing/explaining mental health difficulties and treatment with service users from ethnic minority groups?

- *Did this differ depending on ethnicity?*
- *Did you make any adaptations?*
- *Psychoeducation*

- *Diagnoses*

Are there any identifiable cultural differences when discussing mental health with service users from ethnic minority groups?

- *Social norms, traditions, knowledge, beliefs, laws and habits*
- *What kind of cultural differences exist?*

What barriers exist for service users from ethnic minority groups seeking mental health support?

- *Differences in mental health difficulties – ethnic minority groups and non-minority groups*
- *Reason for differences – cultural differences (patient-centred), lack of cultural sensitivity (service-focused)*

How do these barriers differ from those for service users from non-minority groups?

How did you overcome these barriers?

- *Adaptations*
- *Service users' response to adaptations*

What adjustments have you previously made to accommodate service users from ethnic minority backgrounds?

- *Making information more accessible*
- *Visual aids*
- *Culturally-relevant terms*

Part 3 – Treatment

Did language barriers create an issue during treatment?

- *How were language barriers overcome?*
- *Interpreters/translators*
- *Family member or friend present*

Did these adjustments affect the length of your sessions with service users from ethnic minority groups?

How did these adjustments impact service users?

- *Service users' response to adjustments*
- *Was treatment more accessible/suitable?*

Are there particular characteristics that make a service user more open to seeking mental health support/staying committed to treatment?

- *Generation*
- *Age*
- *Gender*

Is there anything that mental health services could work towards in order to better support service users from ethnic minority backgrounds?

Would you like to add anything else?

- Inform clinician that interview is over
- Thank clinician for participating
